# Sleep disturbance exacerbates atherosclerosis in type 2 diabetes mellitus

**DOI:** 10.3389/fcvm.2023.1267539

**Published:** 2023-12-01

**Authors:** Bingge Fan, Ting Tang, Xiao Zheng, Haixia Ding, Peng Guo, Hongqing Ma, Yu Chen, Yichao Yang, Lihui Zhang

**Affiliations:** ^1^ Department of Endocrinology, The Forth Hospital of Hebei Medical University, Shijiazhuang, China; ^2^ Department of War and Rescue Medicine Field Internal Medicine Teaching and Research Office, NCO School, Army Medical University, Shijiazhuang, China; ^3^ Department of Orthopedics, The Affiliated Hospital, NCO School of Army Medical University, Shijiazhuang, China; ^4^ Department of Endocrinology, The Forth Hospital of Hebei Medical University, Shijiazhuang, China; ^5^ Department of Orthopedics, The Forth Hospital of Hebei Medical University, Shijiazhuang, China; ^6^ Second Department of General Surgery, The Forth Hospital of Hebei Medical University, Shijiazhuang, China; ^7^ Department of Cardiology, Bethune International Peaceful Hospital, Shijiazhuang, China; ^8^ Department of Gastroenterology, Baoding First Central Hospital, Baoding, China; ^9^ Department of Endocrinology, The Second Hospital of Hebei Medical University, Shijiazhuang, China

**Keywords:** sleep disorders, type 2 diabetes mellitus, atherosclerosis, peripheral arterial, smart wristband

## Abstract

**Background:**

Short sleep duration and poor sleep quality are important risk factors for atherosclerosis. The use of smart bracelets that measure sleep parameters, such as sleep stage, can help determine the effect of sleep quality on lower-extremity atherosclerosis in patients with type 2 diabetes.

**Objective:**

To investigate the correlation between sleep disorders and lower-extremity atherosclerosis in patients with type 2 diabetes.

**Methods:**

After admission, all patients were treated with lower-extremity arterial ultrasound and graded as having diabetic lower-extremity vascular lesions according to the results. A smart bracelet was used to obtain the patient sleep data. The correlation between sleep patterns and diabetic lower-extremity atherosclerosis, diabetic foot, and various metabolic indices was verified.

**Results:**

Between August 2021 and April 2022, we screened 100 patients with type 2 diabetes, with 80 completing sleep monitoring. Univariate ordered logistic regression analysis indicated that patients with a sleep score below 76 (OR = 2.707, 95%CI: 1.127–6.488), shallow sleep duration of 5.3 h or more (OR=3.040, 95 CI: 1.005–9.202), wakefulness at night of 2.6 times or more (OR = 4.112, 95%CI: 1.513–11.174), and a deep sleep continuity score below 70 (OR = 4.141, 95%CI: 2.460–615.674) had greater risk of high-grade lower limb atherosclerosis. Multivariate ordinal logistic regression analysis revealed that the risk of high-grade lower limb atherosclerosis was higher in patients with 2.6 or more instances of nighttime wakefulness (OR = 3.975, 95%CI: 1.297–12.182) compared with those with fewer occurrences. The sleep duration curve of patients with different grades of diabetic lower-extremity atherosclerosis was U-shaped. According to the results of the one-way analysis of variance, the higher the deep sleep continuity score, the lower the Wagner scale score for diabetic foot (*P* < 0.05).

**Conclusions:**

Sleep disorders (long, shallow sleep duration, frequent wakefulness at night, and poor continuity of deep sleep) can worsen lower limb atherosclerosis in patients with type 2 diabetes. This finding can provide a new method for medical professionals to prevent and treat diabetic lower-extremity vascular lesions.

## Introduction

Lower extremity atherosclerosis is a common complication of diabetes that can progress to ischemic foot lesions and endanger the lives of patients. The prevalence of foot ulcers ranges from 4%–10%, with an annual population-based incidence of 1.0%–4.1%, and a lifetime incidence as high as 25%. Diabetic ischemic foot disease is the most common cause of nontraumatic amputation (1). Diabetic foot is characterized by treatment difficulties, high disability and fatality rates, and high treatment costs. Collectively, these factors result in physical and mental distress for patients, contributing to a huge social and economic burden. An early understanding of the risk factors for diabetic lower-extremity atherosclerosis is important for the prevention and treatment of ischemic disease in diabetic foot.

Recently, sleep disorders have received increasing attention in the prevention and treatment of vascular diseases. Sleep deprivation has been shown to increase the risk of atherosclerosis (2, 3). Non-apnea sleep disorders are also risk factors for peripheral artery disease. Sleep fragmentation increases oxidative stress, which leads to vascular dysfunction and atherosclerosis (4). Therefore, sleep disorders are irrefutably associated with atherosclerosis. Combined with our clinical observations, we found that the proportion of patients with both sleep disorders and diabetic lower-extremity vascular lesions increased significantly. However, there are few relevant studies, and there is a lack of evidence. Although sleep is known to have an impact on the body, research on this subject has been difficult.

The traditional polysomnographic sleep monitor has limitations in monitoring patients’ long-term sleep status, and the sleep score is a patient recall index that is subject to various interference factors and lacks reliability. To address these issues, we used smart bracelet monitoring to collect sleep data and explore the correlation between sleep patterns and diabetic lower-extremity atherosclerosis. With the advancement of science and technology, the monitoring of daily life signs is easier to be monitored. Smart bracelets are small and convenient, and are gradually recognized by the public. It can record data and save in real time, which significantly improves the patient's compliance. Therefore, as a record requipment, the bracelet is one of the excellent choices.

## Methods

### Study design and patients

A smart bracelet was used to monitor sleep indicators and analyze the correlations between sleep index, diabetic lower-extremity atherosclerosis, and diabetic foot Wagner grade. Patients aged 18 years or older with type 2 diabetes who were admitted to the Department of Endocrinology, Fourth Hospital of Hebei Medical University, between August 2021 and April 2022 were invited to participate. All enrolled patients signed an informed consent form, and the study was approved by the Ethics Committee of the Fourth Hospital of Hebei Medical University (ethics batch number 2022KS016). The inclusion criteria were as follows: (1) Aged 18 years or older; (2) Compliance with the 2021 version of the Chinese diabetes prevention and treatment guidelines. All enrolled patients were required to meet the following diagnostic criteria: presence of typical diabetes symptoms (excessive thirst, polyuria, overeating, unexplained weight loss) + fasting blood glucose ≥ 7.0 mmol/L, or 2-h post-prandial blood glucose ≥ 11.1 mmol/L, or random blood glucose ≥ 11.1 mmol/L. The following categories of patients were excluded: (1) alcoholic patients; (2) pregnant and lactating women; (3) patients with acute exacerbation of chronic obstructive pulmonary disease; (4) patients with sleep apnea; (5) patients suffering from severe organ failure such as heart failure; (6) patients with mental impairments; (7) patients with painful conditions such as diabetic painful neuropathy; (8) patients with severe hypertension (BP > 180/110 mmHg); (9) patients who had recently taken sleep aids; (10) patients with pain score greater than or equal to 1. The exclusion criteria were used to minimize variations in sleep duration and quality due to other medical conditions.

### Procedures

All patients underwent measurements of height, weight, abdominal circumference, hip circumference, blood pressure, and other general indicators. Fasting blood glucose, blood lipid, homocysteine, glycosylated hemoglobin, insulin, and other tests were conducted simultaneously. During hospitalization, lower-extremity arterial ultrasonography or arteriography and ankle-brachial index (ABI) of the lower extremities were performed. Based on the ultrasound results, diabetic lower limb atherosclerosis was divided into four grades. Normal blood vessels without atherosclerotic manifestations were graded as 1. Thickened intima-media thickness without obvious plaque formation was graded as 2. The formation of arterial plaque without significant stenosis (narrowing rate of the artery diameter <20%) was graded as 3. Arterial diameter stenosis ≥20% was graded as 4. The final grade was based on the most severe side. All patients underwent Wagner grading for diabetic foot disease, graded on a six-point scale. The presence of risk factors for foot ulcers without ulcers was graded as 0. A superficial ulcer with no co-infection was graded as 1. A deep ulcer, with the infection limited to the soft tissue, and no bone infection or deep abscess was graded as 2. A deeper infection, which could lead to infection of the bone and deep abscesses, was graded as 3. Grade 4 is localized gangrene, and Grade 5 is gangrene of the entire foot. Professional physicians administered the Pittsburgh Sleep Score (PSQI), Hospital Anxiety and Depression Scale (HADS), and Activity of Daily Living score (ADL) questionnaire.

### Smart bracelet monitoring

Twenty-four hours after hospitalization, a qualified physician placed a bracelet 2 cm above the horizontal line on the patient's left wrist, ensuring it remained close to the patient's skin for over 72 h. The bracelet was inspected by a specialist once a day to ensure the patient wore it correctly and that the battery capacity of the bracelet was sufficient. The bracelet automatically collected data for at least 72 h and generated data analysis charts. The sleep monitoring report recorded the sleep onset, end time, wake time, proportion of deep sleep, proportion of shallow sleep, proportion of rapid eye movement, continuity of deep sleep, wakefulness duration, and breathing quality.

### Statistical analysis

Statistical analyses were performed using IBM SPSS Statistics for Windows, version 26.0. Statistical significance was set at *P* < 0.05. First, Spearman correlation analysis was used to screen the risk factors for diabetic lower-extremity vascular lesions and diabetic foot. Subsequently, the correlation between sleep indices, diabetic lower extremity vascular lesions, and the Wagner scale for diabetic foot was further analyzed using one-way analysis of variance, and ordered logistic regression analysis was used to further clarify the causal relationship between sleep indicators, diabetic lower-extremity atherosclerosis, and the Wagner scale for diabetic foot.

## Results

**Figure 1:**
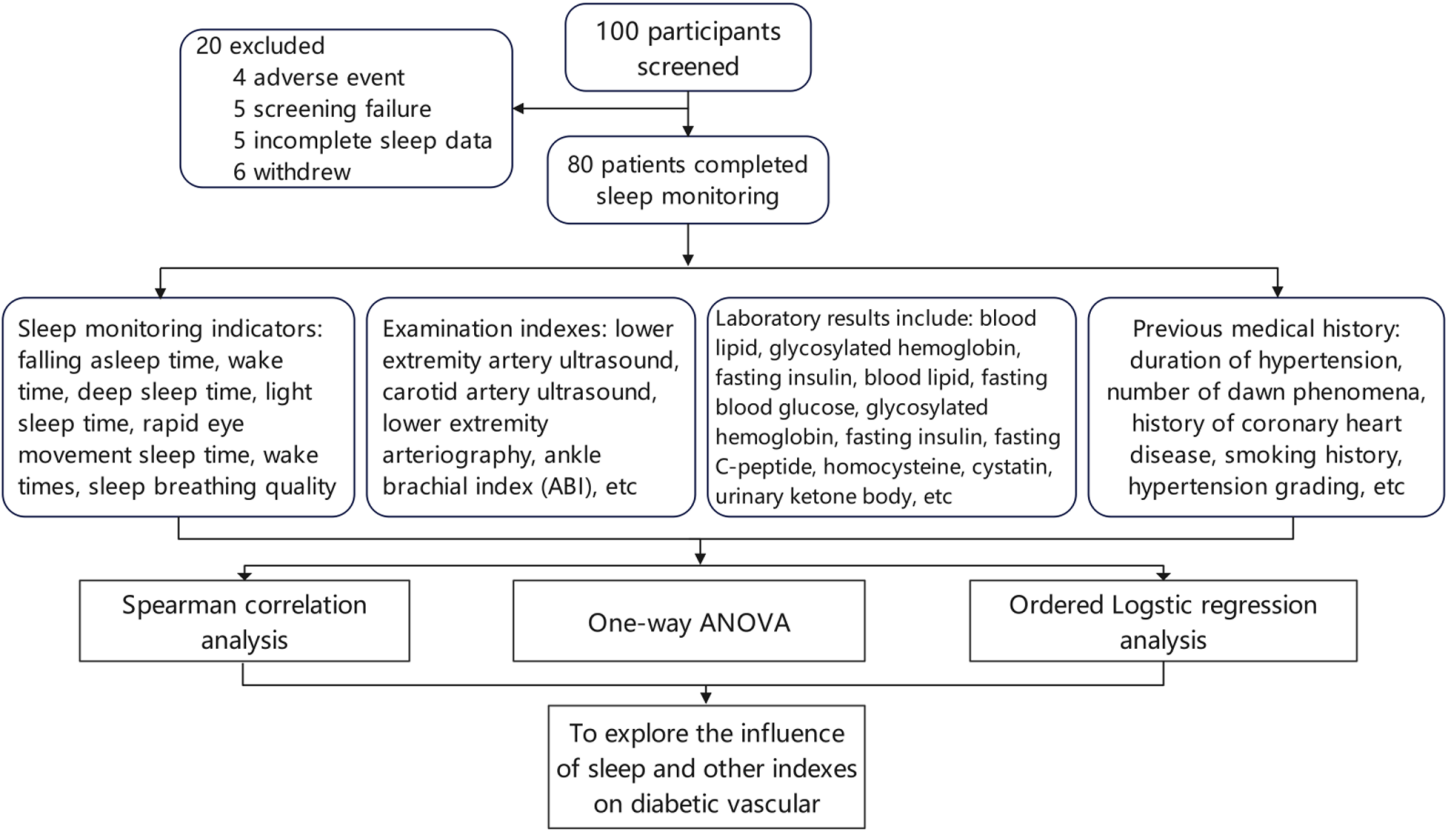
Process technology roadmap. One hundred patients with type 2 diabetes were enrolled after screening, including 41 men and 39 women, of whom 80 patients had complete sleep data. The experimental procedure is shown in the figure.

One hundred patients with type 2 diabetes were enrolled after screening, of whom 80 patients had complete sleep data, including 41 men and 39 women. The experimental procedure is illustrated in (Figure 1). The general characteristics of patients in the different diabetic lower-extremity arteriosclerosis grade groups are shown in (Table 1).

**Table 1:** Baseline of patients with different grades of lower extremity vascular disease.

Project	Grade 1	Grade2	Grade3	Grade4	*F*-value	*P*-value
	(*n* = 18)	(*n* = 9)	(*n* = 43)	(*n* = 10)		
Age (years)	45.94 ± 11.72	57 ± 11.10	64.88 ± 12.73	72.6 ± 7.47	14.81	0.000
Male/female(cases)	8/10	3/6	24/19	6/4	0.70	0.554
Smoking history (years)	1.11 ± 4.71	2.22 ± 6.67	7.28 ± 12.10	11.0 ± 20.79	2.06	0.113
Daily smoking quantity	0.56 ± 2.36	4.44 ± 13.33	7.02 ± 14.08	4.00 ± 6.99	1.34	0.268
Duration of diabetes (years)	4.51 ± 5.33	9.39 ± 6.60	11.78 ± 9.83	17.4 ± 15.75	4.28	0.008
Drinking history (years)	2.11 ± 7.21	4.44 ± 8.82	4.40 ± 10.74	11.0 ± 20.79	0.79	0.504
Daily alcohol consumption(g)	19.44 ± 62.16	62.5 ± 176.78	39.89 ± 229.53	4.00 ± 12.65	0.21	0.891
Length of abstinence (years)	0.22 ± 0.94	0.00 ± 0.00	0.34 ± 1.63	0.00 ± 0.00	0.30	0.828
History of hypertension (years)	2.12 ± 5.09	4.23 ± 5.80	9.25 ± 11.40	9.72 ± 6.24	3.06	0.033
Hypertension grading	0.83 ± 1.15	1.78 ± 1.39	1.58 ± 1.30	2.40 ± 0.70	3.81	0.013
History of coronary heart disease (years)	0.69 ± 1.78	2.44 ± 3.84	2.74 ± 8.09	3.70 ± 61.9	0.56	0.642
History of cerebral infarction (years)	1.11 ± 4.71	1.44 ± 4.33	0.42 ± 1.35	1.20 ± 3.80	0.46	0.713
Abdominal circumference (cm)	92.22 ± 12.44	95.33 ± 11.62	92.91 ± 9.08	95.40 ± 6.98	0.36	0.781
Hip circumference(cm)	99.78 ± 10.60	99.78 ± 7.58	98.47 ± 8.75	100.0 ± 5.23	0.16	0.920
Waist-to-hip ratio	0.92 ± 0.07	0.95 ± 0.05	0.94 ± 0.06	0.95 ± 0.05	0.86	0.466
Height (cm)	166.5 ± 7.09	164.4 ± 9.66	164.9 ± 11.49	167.30 ± 9.13	0.25	0.861
Body weight (Kg)	76.00 ± 13.02	75.33 ± 17.92	71.76 ± 12.96	68.05 ± 7.39	1.00	0.398
BMI (Kg/m2)	27.53 ± 5.40	27.56 ± 4.21	25.73 ± 3.55	24.35 ± 2.39	1.92	0.134
Total cholesterol (mmol/l)	4.62 ± 1.18	4.39 ± 1.67	4.55 ± 1.14	3.82 ± 1.04	1.13	0.344
Triglyceride (mmol/l)	1.96 ± 1.76	1.48 ± 0.74	1.89 ± 1.53	1.33 ± 0.66	0.64	0.593
High-density lipoprotein Cholesterol (mmol/l)	1.20 ± 0.23	1.22 ± 0.25	1.20 ± 0.31	1.03 ± 0.17	1.17	0.328
Glycosylated Hemoglobin	8.20 ± 1.77	7.20 ± 1.04	8.94 ± 1.94	9.07 ± 2.10	2.77	0.047
Fasting insulin (mIU/l)	15.15 ± 10.90	19.29 ± 13.33	11.03 ± 7.18	12.91 ± 15.28	1.78	0.160
Fasting blood glucose (mmol/l)	10.37 ± 4.82	7.24 ± 1.77	9.30 ± 4.61	9.98 ± 6.33	0.95	0.419
Insulin resistance index	6.20 ± 3.07	6.31 ± 5.64	3.95 ± 3.26	5.00 ± 4.15	1.84	0.150
Fatty liver [cases (%)]	4 (22.2)	1 (11.1)	14 (32.6)	4 (40.0)	1.18	0.324
Very low density lipoprotein Cholesterol (mmol/l)	0.32 ± 0.28	0.40 ± 0.35	0.49 ± 0.56	0.38 ± 0.28	0.62	0.602
Apolipoprotein A(g/l)	1.25 ± 0.15	1.38 ± 0.25	1.25 ± 0.22	1.09 ± 0.19	2.96	0.038
Apolipoprotein B(g/l)	0.87 ± 0.24	0.85 ± 0.43	0.91 ± 0.33	0.78 ± 0.33	0.42	0.743
Homocysteine (*μ*mol/l)	11.03 ± 2.32	12.32 ± 3.81	13.41 ± 7.75	13.69 ± 6.74	0.56	0.644
Cystatin (mg/l)	0.63 ± 0.09	0.71 ± 0.13	1.35 ± 1.89	2.14 ± 3.20	1.41	0.250
Wagner scale for Diabetic feet	0.17 ± 0.71	0.00 ± 0.00	0.60 ± 1.42	2.20 ± 2.04	6.24	0.001
Ketosis [cases (%)]	1 (5.5)	0 (0)	6 (14.0)	0 (0)	2.77	0.048
Urinary albumin Excretion rate (%)	0.06 ± 0.24	0.00 ± 0.00	0.12 ± 0.32	0.10 ± 0.32	0.50	0.681
Peripheral neuropathy [cases (%)]	18 (100)	2 (22.2)	24 (55.8)	10 (100)	7.84	0.000
EF (%)	64.5 ± 4.00	64.44 ± 2.46	63.12 ± 6.54	62.20 ± 7.66	0.44	0.723
ABI left posterior tibia (PT)	1.12 ± 0.45	1.10 ± 0.28	1.00 ± 0.21	0.61 ± 0.46	4.72	0.005
ABI right posterior tibia (PT)	1.25 ± 0.31	1.15 ± 0.27	1.01 ± 0.24	0.44 ± 0.51	12.66	0.000
ABI left dorsal foot (DP)	1.12 ± 0.18	1.09 ± 0.28	0.97 ± 0.25	0.62 ± 0.32	7.39	0.000
ABI right dorsal foot (DP)	1.11 ± 0.19	1.17 ± 0.28	1.05 ± 0.18	0.52 ± 0.58	9.93	0.000
Carotid atherosclerosis grading	1.53 ± 0.87	1.86 ± 0.69	2.85 ± 0.62	3.00 ± 0.00	18.27	0.000
ADL score	100 ± 0.00	99.44 ± 1.67	97.68 ± 8.45	94.38 ± 12.37	1.21	0.312
HADS scale score	3.11 ± 5.35	2.75 ± 3.23	3.70 ± 6.04	4.50 ± 3.63	0.21	0.890

### Screengrab of sleep data monitored by the bracelet

**Figure 2:**
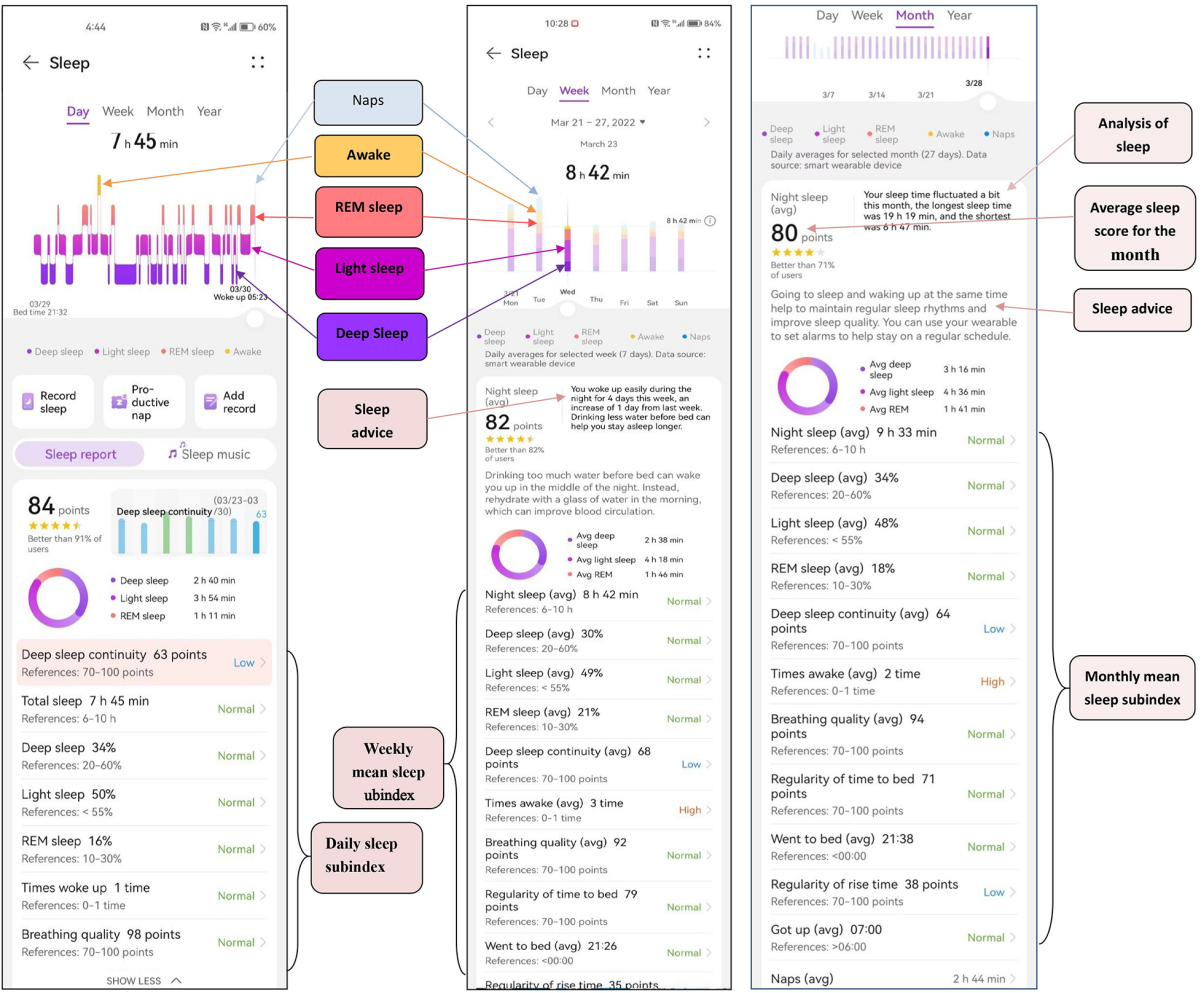
Screengrab of sleep data monitored by the bracelet. Daily, weekly and monthly sleep statistics were monitored by the bracelet.

Daily, weekly and monthly sleep statistics were monitored by the bracelet (Figure 2).

### Analysis of correlative factors of lower extremity vascular atherosclerosis in diabetic patients

The wake time was recorded as 1,440 min (24 h) + wake time (min). Results from the one-way analysis of variance suggested that age, duration of diabetes, duration of hypertension, grade of hypertension, apolipoprotein A level, glycosylated hemoglobin level, Wagner scale for diabetic feet, and ABI were factors related to diabetic lower-extremity atherosclerosis (*P* < 0.05, Table 1). The longer the time of awakening at night, the more serious the diabetic lower-extremity atherosclerosis (*P* < 0.05, Table 2).

**Table 2:** Sleep results recorded by smart bracelets of enrolled patients.

Project	Grade1	Grade2	Grade3	Grade4	*F*-value	*P*-value
	(*n* = 18)	(*n* = 9)	(*n* = 43)	(*n* = 10)		
The score recorded by the bracelet	78.03 ± 3.47	72.81 ± 7.56	73.81 ± 9.8	72.89 ± 4.68	1.510	0.219
Sleep time (min)	1,342.2 ± 86.26	1,317.8 ± 153.69	1,306.4 ± 62.30	1,256.7 ± 81.54	2.265	0.088
Awakening time +1,440 (min)	1,831.5 ± 30.28	1,814.0 ± 56.24	1,814.6 ± 104.99	1,884.9 ± 234.67	1.066	0.369
Total sleep duration (h)	7.26 ± 1.09	6.29 ± 0.98	7.77 ± 1.78	8.26 ± 3.01	2.380	0.076
Deep sleep duration (h)	1.96 ± 0.48	1.76 ± 0.24	2.13 ± 0.71	2.37 ± 0.84	1.738	0.167
Duration of shallow sleep (h)	3.95 ± 0.74	3.50 ± 1.13	4.58 ± 1.59	5.31 ± 1.71	3.565	0.018
Rem sleep duration (h)	1.35 ± 0.37	1.03 ± 0.46	1.22 ± 0.50	1.33 ± 0.57	1.015	0.391
Proportion of deep sleep (%)	26.78 ± 3.47	28.64 ± 5.12	27.42 ± 6.41	23.74 ± 8.95	1.225	0.307
Proportion of shallow sleep (%)	54.37 ± 5.87	54.49 ± 10.71	57.78 ± 10.56	54.83 ± 21.29	0.525	0.666
Percentage of REM sleep (%)	18.67 ± 4.73	16.84 ± 7.61	16.13 ± 6.00	14.52 ± 10.01	1.016	0.391
Deep sleep continuity score	62.16 ± 9.58	61.33 ± 8.85	60.70 ± 8.33	59.46 ± 7.15	0.247	0.863
Night sober times	1.79 ± 0.89	2.88 ± 0.93	3.36 ± 2.07	4.08 ± 2.22	4.611	0.005
Respiratory quality	92.51 ± 0.59	91.65 ± 10.09	92.10 ± 9.61	90.09 ± 12.38	0.180	0.910

**Figure 3:**
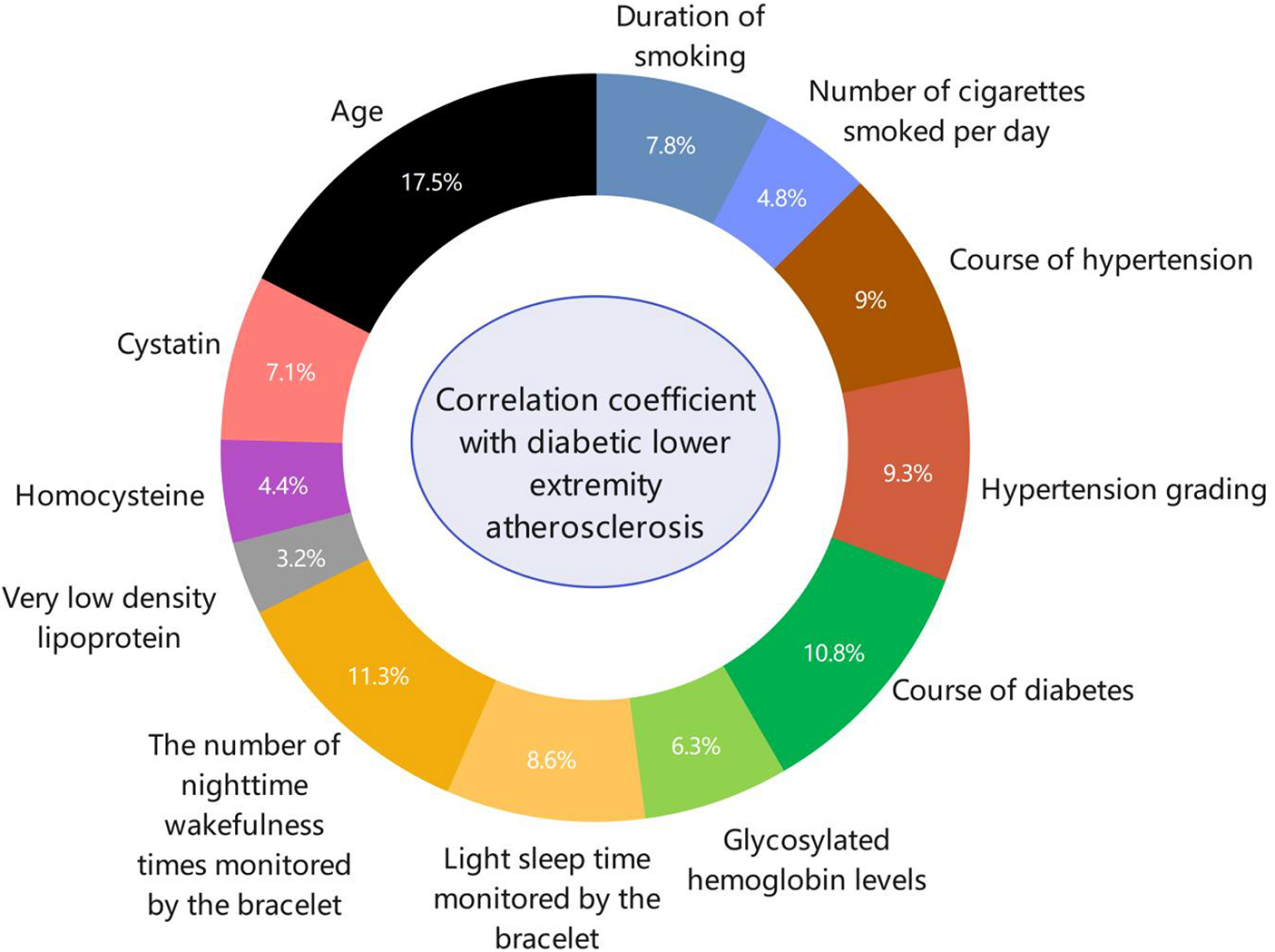
The ring-shaped proportion of risk factors for diabetic lower-extremity vascular disease. Spearman correlation analysis was used to analyze the correlation factors of diabetic lower-limb atherosclerosis. The correlations in descending order were age, number of night awakenings recorded by the bracelet, diabetes course, hypertension grade, hypertension course, light sleep duration recorded by the bracelet, age at smoking, cystatin, glycosylated hemoglobin level, average daily smoking amount, homocysteine level, and very low-density lipoprotein level.

Spearman's correlation analysis was used to analyze the correlation between the sleep data monitored by the bracelet, the classification of diabetic lower-extremity vascular lesions, and other general indicators. The correlation coefficients ranked from high to low were age, number of night awakenings recorded by the bracelet, diabetes course, hypertension grade, hypertension course, shallow sleep duration recorded by the bracelet, age of smoking, cystatin level, glycosylated hemoglobin level, average daily smoking amount, homocysteine levels, and very low-density lipoprotein levels (Figure 3).

**Figure 4:**
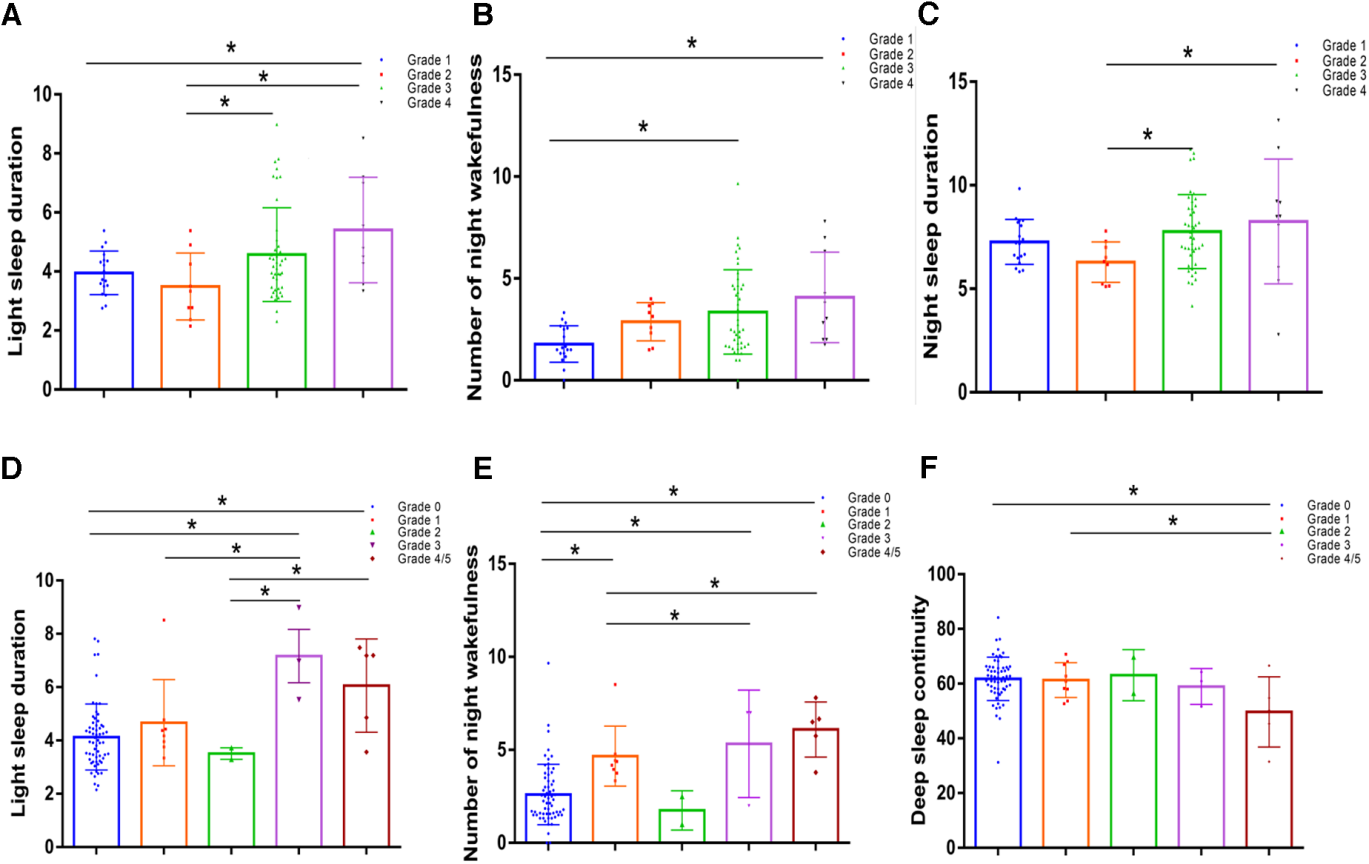
Comparison of sleep indices in patients with different diabetic lower limb atherosclerosis grades and different diabetic foot wagner grades. (**A**) Comparison of night awakenings in patients with different grades of lower-extremity arteriosclerosis. (**B**) Comparison of light sleep duration in patients with different grades of lower extremity arteriosclerosis. (**C**) The sleep duration curve of patients with different grades of diabetic lower-extremity atherosclerosis was U-shaped. (**D**) Comparison of light sleep duration in patients with different Wagner grades of diabetic feet. (**E**) Night awakenings in patients with different Wagner grades of diabetic feet. (**F**) Comparison of deep sleep continuity between patients with different Wagner scores. Values represent the mean and standard deviation (*n* = 80).

One-way analysis of variance was used to compare the differences in shallow sleep duration among the different diabetic lower limb atherosclerosis groups. The results suggested that the longer the shallow sleep duration, the higher the overall trend in diabetic lower-limb atherosclerosis grade (*P* < 0.05, Figure 4A). Additionally, the greater the number of night awakenings, the higher the grade of diabetic lower-limb atherosclerosis (*P* < 0.05, Figure 4B). The sleep duration curve of patients with different grades of diabetic lower extremity atherosclerosis was U-shaped (Figure 4C).

Thirteen Sleep variables were successively included in the univariate ordered logistic regression, and it was found that four variables were correlated with diabetic lower extremity vascular disease, including sleep scores, shallow sleep duration, number of night awakenings, and deep sleep continuity. Patients with a sleep score of below 70 (OR = 2.707, 95% CI: 1.127–6.488), shallow sleep duration exceeding 5.3 h (OR = 3.040, 95% CI: 1.005–9.202), more than 2.6 times occurrences of wakefulness at night (OR = 4.112, 95% CI: 1.513–11.174), and a deep sleep continuity score below 70 (OR = 4.141, 95% CI: 2.460–615.674) had a greater risk of high-grade lower limb atherosclerosis. (*P* < 0.05, Table 3).

**Table 3:** Results of univariate ordinal logistic model using 4 levels of diabetic lower extremity vascular disease as response.

Variable	Level	*β*	OR	95%CI	*P*-value
Sleep scores	<76	0.996	2.707	1.127-6.488	0.026
	≥76	Ref			
Duration of shallow sleep	≥5.3	1.112	3.040	1.005-9.202	0.049
	<5.3	Ref			
Number of night wakefulness	≥2.6	1.414	4.112	1.513-11.174	0.006
	<2.6	Ref			
Deep sleep continuity	<70	1.421	4.141	2.460-15.674	0.036
	≥70	Ref			

Four significant predictors of diabetic lower extremity vasculopathy from the univariate analysis were included in the multiple ordinal logistic models. In the final model, only one variable emerged as a statistically significant independent determinant of the severity of diabetic lower extremity atherosclerosis. Compared with patients with an average of less than 2.6 nighttime awakenings, patients with 2.6 or more awakenings had a higher (OR = 3.975, 95% CI: 1.297–12.182) risk of increased classification of diabetic lower extremity vascular disease (*P* < 0.05, Table 4).

**Table 4:** Results of multiple ordinal logistic model using 4 levels of diabetic lower extremity vascular disease as response.

Variable	Level	β	OR	95%CI	*P*-value
Number of night wakefulness	≥2.6	1.380	3.975	1.297–12.182	0.016
	<2.6	Ref			
Sleep scores	<76	0.445	1.560	0.610–3.990	0.353
	≥76	Ref			
Duration of shallow sleep	≥5.3	0.720	2.054	0.454–6.488	0.220
	<5.3	Ref			
Deep sleep continuity	<70	1.333	3.792	0.914–15.737	0.067
	≥70	Ref			

### Analysis of related factors of diabetic foot

Because the number of patients with diabetic foot grades 4 and 5 was small, the statistical analyses of grades 4 and 5 were combined as grade 4. According to the results of a one-way analysis of variance, the higher the deep sleep continuity score, the lower the Wagner scale score for diabetic foot (*P* < 0.05, Figure 4F). The shallow sleep and night wake times of patients with different Wagner grades of diabetic foot did not show a significant step-change trend (Figures 4D,E), which may be related to the small number of cases, most of which were grade 0.

## Discussion

There is growing evidence that people with peripheral artery disease are at increased risk of other health complications, including cardiovascular death, stroke, heart failure, and myocardial infarction (5). Diabetic lower-extremity atherosclerosis can also progress into diabetic foot, which is one of the most serious complications of diabetes. If diabetic foot is not treated promptly, severe cases can lead to amputation and even death (6). This disease has a high disability rate, mortality rate, and huge treatment costs, causing physical and mental pain in patients and adding to the social and economic burden (7). This has become an urgent issue that needs to be addressed.

Studies have linked sleep disorders with diabetes (8). Research suggests that approximately 42%–71% of patients with type 2 diabetes have varying degrees of sleep disorders (9). Many studies have found that lack of sleep increases the incidence of diabetes and aggravates metabolic disorders (10–12). Meanwhile, studies have found that sleep disorders also play an important role in the occurrence, development, and treatment of atherosclerosis, especially cardiovascular and cerebrovascular diseases (13–15). Sleep disorders cause hyperfunction of the sympathetic nervous system and neuroendocrine abnormalities, and promote the secretion of the adrenal hormones melatonin, angiotensin, and catechin, thus leading to vasoconstriction, and increased blood pressure (16), and increased platelet viscosity, thus promoting the progression of vascular lesions (17)_._

Smart wearable devices are increasingly being used in clinical settings. Data generated by intelligent wearable devices are often referred to as “big data,” and various machine algorithms used to predict health outcomes have become an area of common interest in industry, academia, and healthcare research (18). Smart wearable devices have great potential in personal health management and clinical care. It can continuously monitor health data, provide clues for disease diagnosis, discover disease symptoms promptly, and help doctors make comprehensive and accurate judgments. With progress in science and technology, various brands of smart bracelets or watches can monitor the general vital signs of human beings in a noninvasive, continuous, and convenient manner, such as heart rate, oxygen saturation, amount of exercise, blood pressure, and other indicators. However, sleep monitoring remains in its infancy. To address this, we selected a smart bracelet that primarily utilizes cardiopulmonary coupling (CPC) technology through the heart rate and acceleration sensors built into the watch. Chen et al. demonstrated that an accelerometer provided good results in predicting sleep duration, while CPC technology is useful for obtaining data on sleep apnea and assessing sleep conditions for insomnia (19–21). According to the Center for Dynamic Biomarkers, Beth Israel Deaconess Medical Center/Harvard Medical School, the results of sleep state analysis based on TruSleep™ sleep detection technology were highly consistent with the results of cardiopulmonary coupling analysis based on electrocardiography (ECG). The accuracy of comprehensive sleep duration was 93.8%, deep sleep duration was 88.8%, shallow sleep duration was 90.7%, and rapid eye movement (REM) sleep duration was 85.6%. According to verification by the University of Bern in Switzerland, the accuracy of TruSleep™ sleep detection technology for the recognition of sleep state was 96.3% for standard polysomnography monitoring, and the certification results were included in the well-known academic conference of the European Academy of Neurology (EAN) in 2018. In our study, a one-way analysis of variance was used to compare the differences in sleep indicators recorded by the bracelet in different diabetic lower limb atherosclerosis groups. The results showed that the more times of awakening at night, the more severe the diabetic lower-extremity atherosclerosis (*P* < 0.05).

Although studies on diabetic lower-extremity atherosclerosis are relatively comprehensive, studies on the effect of sleep on diabetic lower-extremity atherosclerosis are still insufficient. Suzuki et al. studied the association between sleep fragmentation and human atherosclerosis and found that sleep fragmentation was independently associated with atherosclerosis (2, 22). Nair and Zhang reported that long-term sleep fragmentation leads to vascular dysfunction and atherosclerosis (23). Increased wakefulness is the main manifestation of sleep fragmentation. In our study, the results showed that sleep disorders in patients with diabetes positively correlated with the progression of lower limb atherosclerosis. Univariate ordered logistic regression analysis results suggested that patients with a sleep score below 70, shallow sleep duration exceeding 5.3 h, more than 2.6 times of wakefulness at night, and a deep sleep continuity score below 70 have a greater risk of high-grade lower limb atherosclerosis. Our study showed that long, shallow sleep durations, frequent wakefulness at night, and poor continuity of deep sleep could aggravate atherosclerosis of the lower limbs in patients with diabetes. Meanwhile, our results suggested that the sleep duration curve of patients with different grades of diabetic lower limb atherosclerosis was U-shaped, which is consistent with the results of Beaman et al. (24, 25).

Studies have shown that after Cognitive Behavioral Therapy for insomnia (CBT-I), patients experience significant improvements in HbA1c levels, diabetes self-care behaviors, and fatigue (26). In our study, the results of multivariate ordinal logistic regression analysis showed that compared with patients with an average of less than 2.6 waking times at night, patients with 2.6 or more waking times at night had a 3.975 times higher risk of advanced diabetic lower extremity vascular disease. We found that increased nocturnal wakefulness was an independent risk factor for lower limb atherosclerosis in patients with diabetes. If our findings are applied in the clinical settings, and CBT-I treatment is guided through education for diabetic patients to reduce their nocturnal waking times, this approach may be a new method for preventing diabetic lower limb atherosclerosis.

### Limitation

Our study has some limitations, including insufficient types of bracelets, a relatively small sample size, and a short follow-up period. Additionally, our study was cross-sectional; therefore, the results need to be confirmed in further longitudinal studies.

## Conclusion

We explored the relationship between sleep and diabetic lower-extremity atherosclerosis via a smart bracelet, a modern technological product, using a simple, effective, and rapid method. These results can guide in further understanding of the relationship between sleep and diabetic lower-extremity atherosclerosis. These findings partly demonstrate that sleep disorders (long, shallow sleep, frequent wakefulness at night, and poor continuity of deep sleep) aggravate lower-extremity atherosclerosis in patients with type 2 diabetes. Moreover, this study can provide a new method for medical professionals to prevent and treat diabetic lower-extremity vascular lesions.

## Data Availability

The original contributions presented in the study are included in the article/**Supplementary Material**, further inquiries can be directed to the corresponding author.
